# A digital assistant for shading paper sketches

**DOI:** 10.1186/s42492-020-00049-7

**Published:** 2020-06-08

**Authors:** Amal Dev Parakkat, Hari Hara Gowtham, Sarang Joshi, Ramanathan Muthuganapathy

**Affiliations:** 1 Indian Institute of Technology Madras, Chennai, 600036 India; 2 Indian Institute of Technology Guwahati, Assam, 781039 India

**Keywords:** Shading, Paper sketch, Mixed reality, Iso-contours, Delaunay triangulation, Digital art

## Abstract

We present a mixed reality-based assistive system for shading paper sketches. Given a paper sketch made by an artist, our interface helps inexperienced users to shade it appropriately. Initially, using a simple Delaunay-triangulation based inflation algorithm, an approximate depth map is computed. The system then highlights areas (to assist shading) based on a rendering of the 2.5-dimensional inflated model of the input contour. With the help of a mixed reality system, we project the highlighted areas back to aid users. The hints given by the system are used for shading and are smudged appropriately to apply an artistic shading to the sketch. The user is given flexibility at various levels to simulate conditions such as height and light position. Experiments show that the proposed system aids novice users in creating sketches with impressive shading.

## Introduction

**Fig. 1:**
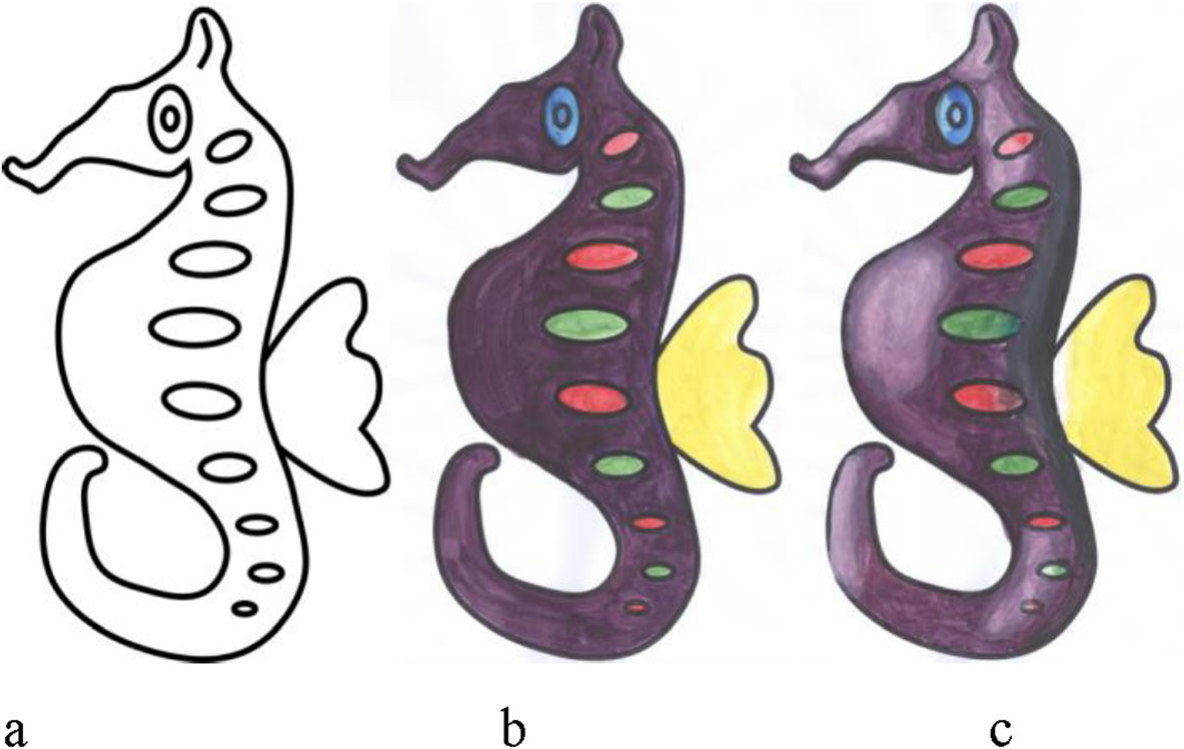
**a**: A sample sketch; **b**: After applying flat coloring; **c**: Coloring with appropriate shades

Given an outer boundary, it is easy to fill the area with a single color. However, shading brings life to a sketch. A plain and simple sketch can be made attractive by shading it appropriately (Fig. 1 shows the result of plain coloring and shading on a sketch). Appropriate shading might motivate the user to become more involved in such activities as it gives a 3-dimensional (3D) feel of the 2-dimensional (2D) sketch. However, most people find it difficult to create an artistic feel through shading. By experiments, we found that the difficulty is not due to lack of ability. Instead, there is a lack of proper knowledge about where to apply which color. In this work, we introduce first of its kind user assistance system to shade a sketch on real paper. To find the essential pieces of information that aid shading, a 3D correspondence has to be inferred from the sketch. Though a lot of work has been done to create a 3D reconstruction from a single image, it is proven that general 3D reconstruction is difficult. This is primarily because of the difficulties in computing the depth map accurately. Fortunately, for applications such as shading, rather than computing absolute depth, it is perhaps sufficient to compute an approximate one. Shading also gives a symmetric 3D feel about the plane of the sketch. Hence, one can view shading as creating a 2.5-dimensional (2.5D) (throughout this paper, we make use of the term 2.5D, to denote an incomplete 3D model which can only be used for faking the depth) feel rather than 3D, eliminating the need to go for computational intensive procedures for computing 3D depth. Such an approach can also lead to a reduced number of user interventions.

Once a 2.5D digital representation of the sketch is calculated, a shader can be applied to provide a 3D look to the model. Replicating this 3D-lookalike shading to a paper sketch (a sketch in the physical world) is not straightforward because of the presence of a large number of pixel colors available in rendered images. One way to address this is to place minute dots in each position corresponding to a pixel in the paper sketch, with the pixel color as in the rendered image. Since this is time-consuming and we may not have all the colors corresponding to each pixel in the rendered image, the task becomes cumbersome. To overcome this, the pixels in the rendered image can be grouped appropriately to make shading easy.

Even though various methods exist to assist the sketching process on a paper sketch, to the best of our knowledge, no previous research has been published to support shading a paper sketch without additional information. In this context, we present an interactive digital assistant whose aim is to create impressive shadings and to make the shading task easier, particularly for novice users. Based on very little information provided by the user, a 2.5D inflation of the digital version of the paper sketch (acquired by scanning) is computed. Since the main factors influencing shading include depth, base color, lighting position, and intensities, the user is given the flexibility to adjust these aspects.

**Fig. 2:**
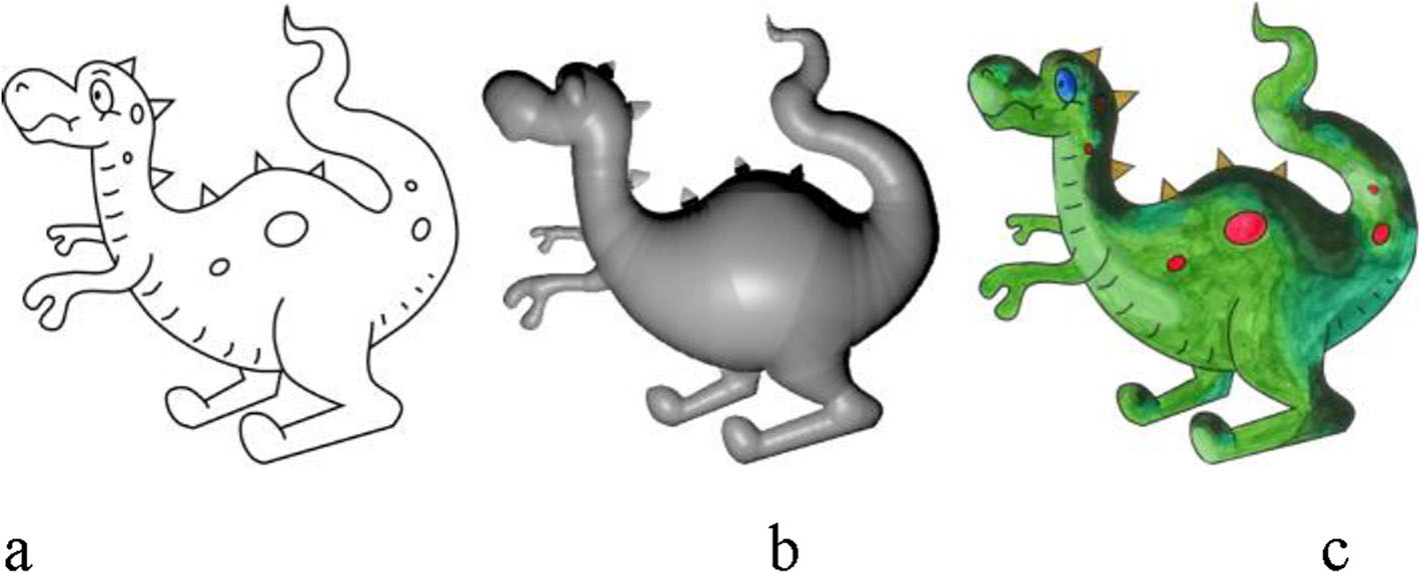
**a**: A sample sketch; **b**: 2.5D inflation mesh; **c**: Shading done with our system assistance

In this paper, the major contribution is our framework, which contains three parts: a 2.5D inflation algorithm, iso-contour computation with appropriate colors, and a mixed reality interface. The major challenges for designing this system can be described as follows: First, we need a simple to understand and implement inflation algorithm to create a 2.5D model from the sketches. Second, user interactions should be easy, intuitive, and straightforward since the target users are novices and may include artists who are not comfortable using complicated user interfaces. Finally, the iso-contours generated should be easy to map into the physical medium (i.e., mapping from digital iso-contours to a paper sketch). The first two challenges are handled by using simple and easy to edit 2.5D modeling. To address the final challenge, we took advantage of the power of the mixed reality interface. Figure 2 shows a sample sketch, an inflated mesh in 2.5D, and the shading given with the assistance of our system.

### Related framework

Software-based assistance used to help novice users create artwork is not a new concept in the computer graphics community. The level of support spans from assisting simple processes like sketching [1] and coloring [2], up to creating complex tasks like clay sculpting [3], making wire arts [4], wind-up toys [5], pop-up paper models [6], generating abstract 3D representations with planar sections [7] or pipes [8]. Computer assisted tools for developing art works like Deco [9], Weavy [10], the design of iris folding patterns [11] are also used to support artistic tasks.

Aside from the non-photo realistic rendering of digital images, some contributions concentrate on using digital assistants to create art in the physical world. For example, Shilkrot et al. [2] introduced a device with 6 degree of freedom tracking to facilitate an augmented airbrush for computer-aided painting. Whereas, Prévost et al. [12] introduced a system which tracks the position of a spray can and determines the amount to be dispersed for replicating an image with spray paint.

Some published research focuses on making the coloring process easy and fun-filled. Two main contributions moving in this direction are by Clark et al. [13] and Magnenat et al. [14]. Both use the power of augmented reality to demonstrate the impact of coloring a sketch by mapping the effect on an associated 3D model in real-time. A connecting-the-dots approach using an augmented reality interface for sketching can be observed in ref. [15]. Various commercial products such as Crayola Color Alive [16], Chromville [17], and Disney’s “Color and Play” [18] are also available which help to visualize the effect of coloring, on an associated 3D model. The main disadvantage of such systems is the need for apriori knowledge about the associated 3D model for each sketch. MagicToon [19] offers another similar publication in which the main objective is to facilitate a 3D color mapped cartoon model creation from 2D drawings. The system creates an automatic 3D model from a 2D sketch and also provides operations to edit and animate the model. Flagg and Rehg [20] help users to imitate the given painting with real-time feedback. The input painting is divided into layers and, with the help of a projector and a real-time camera, the user is asked to paint. Aside from the coloring assistants, manipulating shading based on inflated models have also been presented in refs. [21–23]. Recently Panotopoulou et al. [24] developed a wooden block painting system. Unfortunately, this is not scalable and requires a dedicated set of wooden blocks for each painting. The natural alternative to avoid these limitations is to use an augmented/mixed reality interface. Significant advantages for using such a system include the ability to reuse resources and the cost-effective setup.

## Methods

### Overall framework

**Fig. 3:**
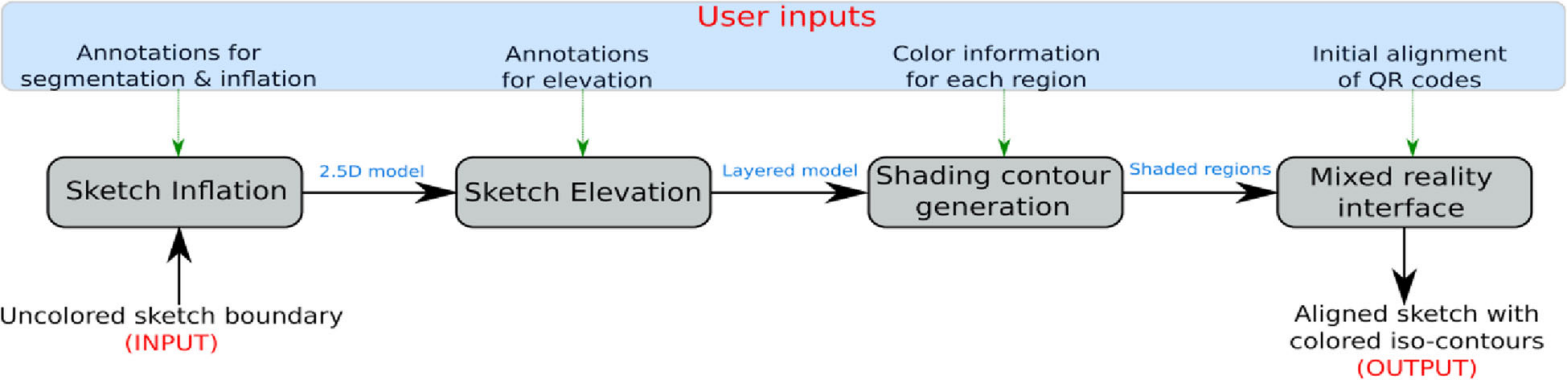
Overall pipeline of the proposed system

Figure 3 shows the overall pipeline of our shading assistance system. The system starts with an uncolored sketch boundary provided by the user, which has yet to be colored. Initially, based on user annotations, the sketch is segmented into different pieces. With the help of the user, the segments are inflated using a Delaunay-triangulation based sketch inflation algorithm and layered appropriately. Toon shading is applied to this layered model to compute various iso-contours such that each iso-contour can be filled with a single color. Using a digitally colored sketch (boundary sketch colored using a flood fill procedure) as a reference, we identify the colors to be filled inside each iso-contour. The shades, along with appropriate regions, are projected back to the table surface with the help of a mixed reality interface. The guidelines (areas along with shades) are used to create beautiful shades on the sketch.

### Sketch inflation

Given a sketch, various algorithms exist for generating 2.5D inflation. Though methods explained in Teddy [25], RigMesh [26] can be used for generating same, we use the following approach for inflation:

- Boundary pixels of the shape to be inflated are extracted and used to create a point set;
- Delaunay triangulation (DT) of the point set is computed, and triangles lying outside the shape are removed (as in ref. [27]);
- Each remaining triangle in the triangulation is inflated using the following procedure:
- The boundary of the triangle is divided into *n* points (*n* is set as 24 for the experimental purpose by applying mid-point subdivision on edges, such that each point can be elevated appropriately to convert the edge into a semi-circle) and let N be the set of all *n* points;
- DT of N is computed;
- Each sampled point in the edges other than the exterior edges (edges which are not part of more than one triangle) is then assigned a z-coordinate based on its distance from the midpoint of the edge, *d*:
  1 $$ {h}_0=e\ast \sqrt{\left(\frac{\left\Vert AB\right\Vert }{2}\right)}2-{d}^2 $$

where A and B are the endpoints of the edge, and e ∈ [− 2, 2] is an elevation parameter used to control the height of inflation.

- Connectivity is made between elevated points based on DT(N)

**Fig. 4:**
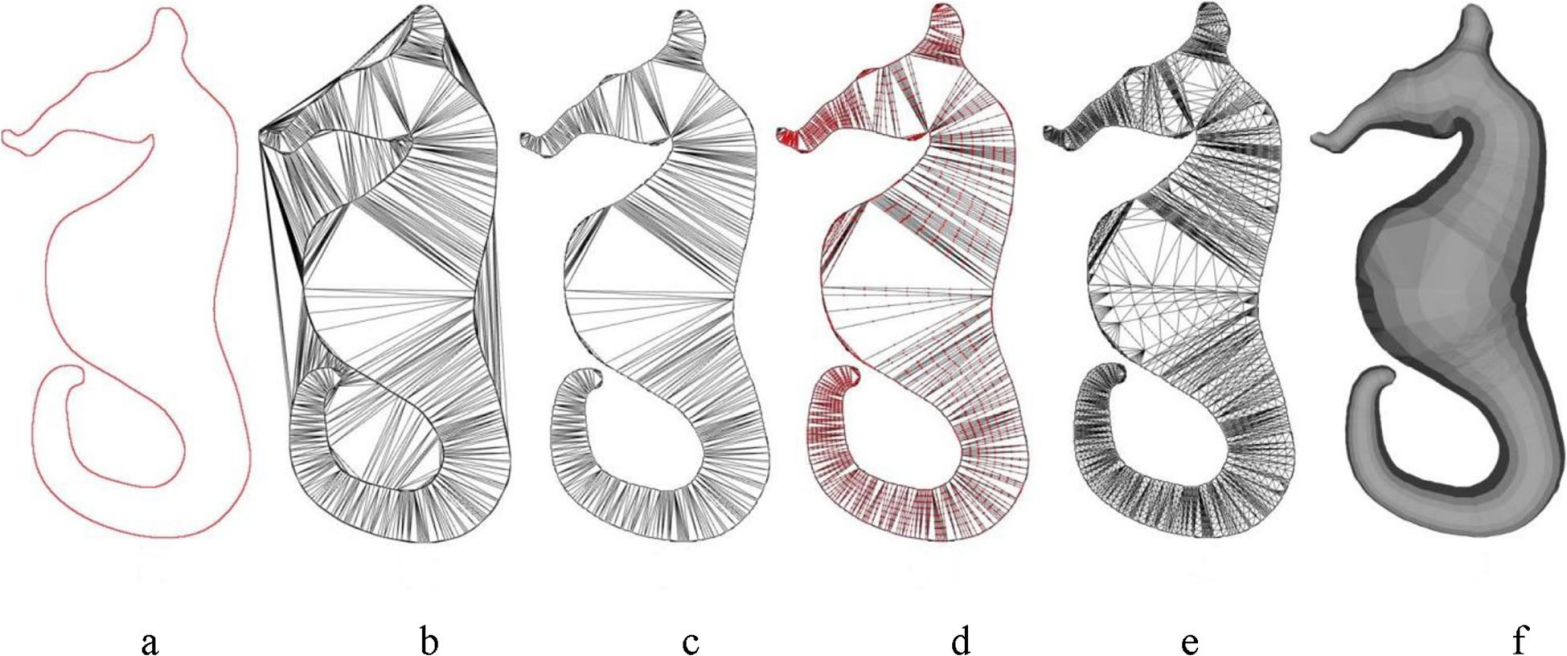
Various steps in the inflation procedure. **a**: A sketch boundary; **b**: DT of the point set extracted from the boundary pixels; **c**: Result after Delaunay sculpting; **d**: Result after edge subdivision; **e**: DT after edge subdivision; **f**: Result after inflation

**Fig. 5:**
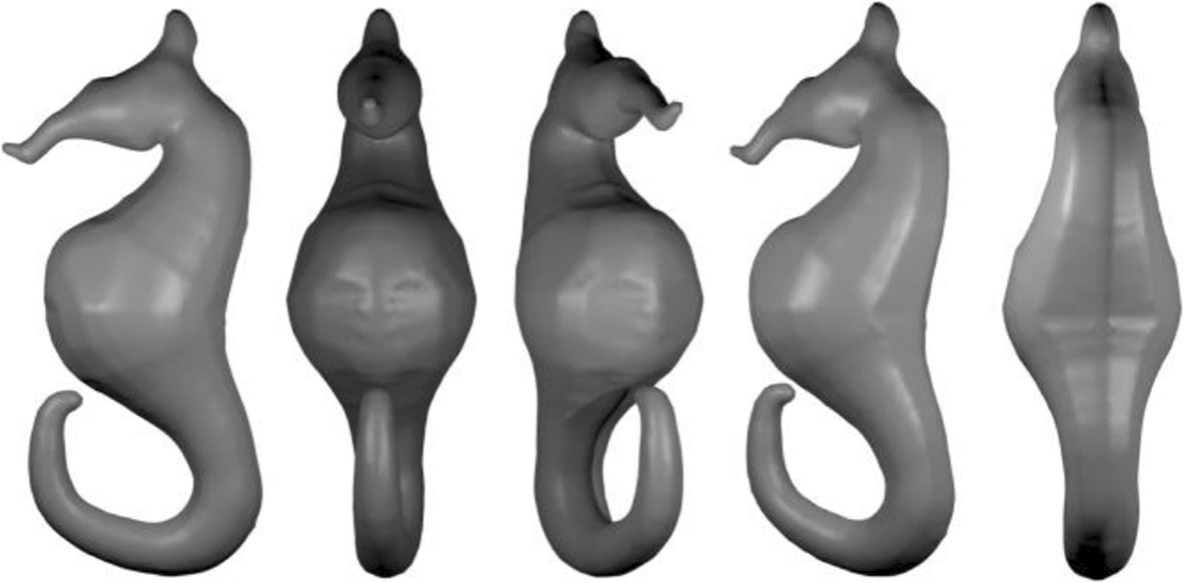
Mirror symmetric 3D model (after uniform resampling) generated by the inflation procedure

Figure 4 demonstrates the various steps in the inflation algorithm. Starting from a simple sketch, as shown in Fig. 4a, pixels are converted into points and used to compute the DT (Fig. 4b), triangles lying outside the shape are removed (Fig. 4c), and the result of edge subdivision is shown in Fig. 4d. The result after computing the DT of each triangle under consideration is shown in Fig. 4e, and Fig. 4f shows the final inflated result after assigning a depth value to each sampled point. It should be noted that assigning positive and negative depth values along with connection information derived from the DT, can be used to create mirror-symmetric 3D models. Figure 5 shows a sample mirror-symmetric 3D model (after smoothing with uniform resampling) generated with the help of our inflation procedure.

Since our input is a plain sketch without any additional information, we apply a number of pre-processing operations to order them. Given a sketch, it is possible to create different 2.5D models because of the presence of textures and different elevation levels. Since the texture, elevation, and layering information cannot be directly inferred from the sketch, we involve user intervention to create the 2.5D model, which best approximates user imagination. To achieve this, we took advantage of interactive region-based segmentation and elevation procedures.

#### Region based segmentation

**Fig. 6:**
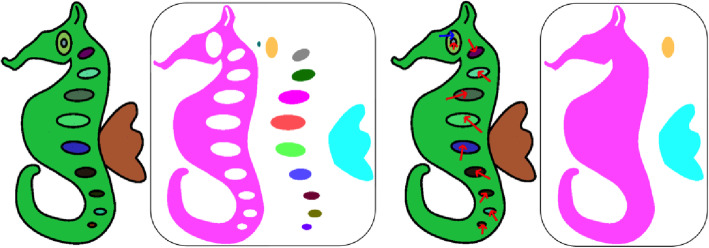
(Left to Right) **a**: Sketch after region based segmentation; **b**: Identified regions; **c**: User annotations for region updation; **d**: Updated regions

Given a sketch, one of the difficult tasks is to segment it meaningfully. Unfortunately, automatic segmentation becomes more challenging if textures are present in the input sketch (the difficulty lies in distinguishing textures from the regular parts). Rather than placing extra constraints on the input sketch, we involve user intervention to complete the task. From a user’s point of view, identifying textures from a sketch is a straight-forward task. Starting from an initial segmentation in which each closed region will be a segment (by repeatedly applying the Flood-Fill algorithm), based on the user annotations, regions are updated. User annotation is applied by marking two regions and defining one of the two operations:

1. Merging regions: Segments corresponding to the selected regions are merged (for example, removal of textures from the body of the seahorse in Fig. 6d). The red arrows in Fig. 6 illustrate this operation, where we merge the regions at the end and tip of the arrow.
2. Separating regions: The regions are separated and considered as two independent regions (for example, separating the eye as a different region from the body of the seahorse in Fig. 6d). The blue arrow in Fig. 6 illustrates this operation, where we separate the regions at the end and tip of the arrow to create two independent regions.

The user can also mark hole regions that do not require inflation. Figure 6a shows a sample sketch after automatic region segmentation and the respective segments (Fig. 6b), the user annotations on automatic segmentation and the segments after user intervention are shown in Fig. 6c and Fig. 6d.

**Fig. 7:**
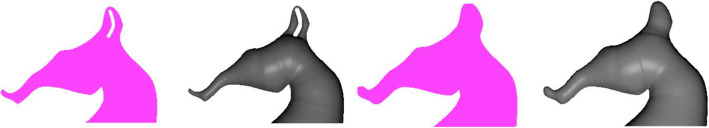
(Left to Right) **a**: Part of a region; **b**: Its inflated result; **c**: Region shown in (**a**) after dilation operation; **d**: Inflated result of (**c**)

Once segmentation is over, the inflation algorithm can be applied for each segment individually. However, the presence of decorative lines (small decorative line in the ear of the seahorse as shown in Fig. 7a) will leave some thin holes in the segmented result. To address this problem, before inflation, we applied a morphological thinning operation on the segmented result, which fills the gap made by decorative lines. Figure 7 shows the effect of the dilation operation on the inflation result.

#### Sketch elevation

**Fig. 8:**
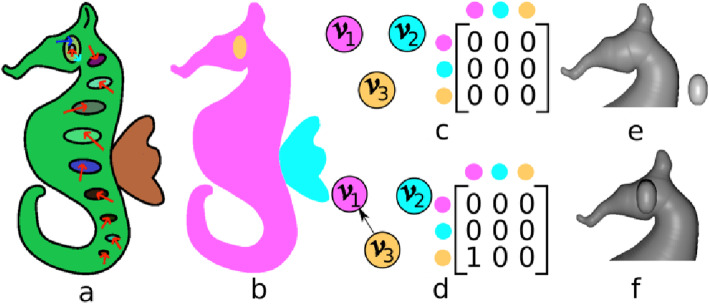
**a**: Annotated sketch along with region elevation marking (cyan colored arrow); **b**: Regions; **c**, **d**: Graphs (**c**) before and (**d**) after region elevation marking; **e**, **f**: Models (**e**) before and (**f**) after region elevation

Another important property that must be considered in a 2.5D model is the relative heights. The inflated regions have to be layered appropriately, and this is a difficult task to automate. In this implementation, we employed user annotations (cyan colored arrows) to give a relative depth setting. Initially, a directed graph G is made with vertex set V, where each v_i_ ∈ V represents a region. As the user annotates, edges are made between appropriate regions (Fig. 8 shows an example of this). Based on G, the height of the 2.5D model of the corresponding region is relatively set. Let vertices v_i_ and v_j_ correspond to regions R_i_ and R_j_ respectively and G has an edge from v_i_ to v_j_, then the inflation of R_j_ is elevated to the projection plane defined by the maximum z-coordinate of R_i_. To avoid confliction, a cycle of regions where one overlaps the other is not allowed.

The following steps are taken to elevate one region over the other:

- We first find the highest z-coordinate at each vertex of the region we are elevating, by creating an intersection between a ray along z-axis passing through the vertex with the thus far obtained 2.5D mesh.
- Among all the intersection points, the highest value is found (say z_0_), and is used as the base height for the elevated region instead of 0, i.e., the value z_0_ is added to the height h_0_, obtained by inflation of the region, as (h_0_) as h(p) = h_0_(p) + z_0_.

Figures 8a-e show an annotated sketch (cyan arrow shows the elevation annotation), regions, initial graph and the graph after introducing the edge. Alongside this are the adjacency matrices, inflation of individual regions, and the final inflated mesh after elevating the eye region.

#### Boundary elevation

**Fig. 9:**
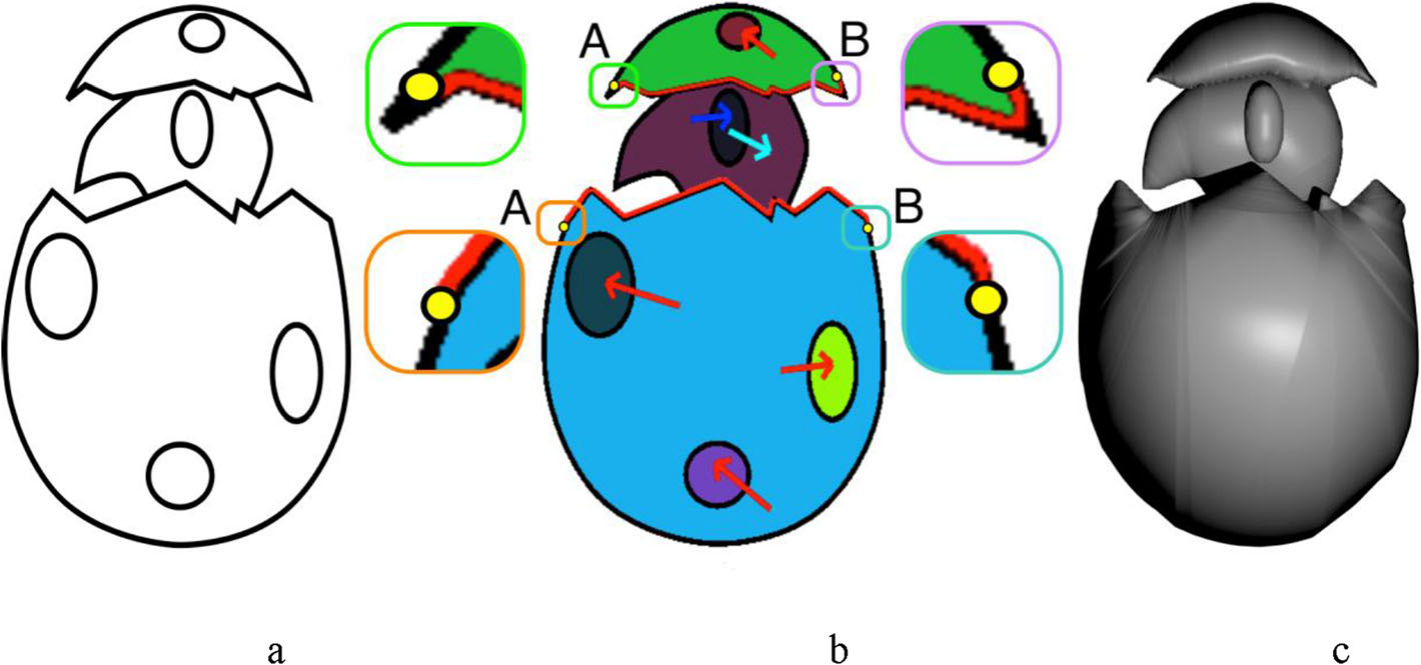
Boundary elevation. **a**: Input sketch; **b**: Annotated flood fill segmentation along with system calculated boundaries to be elevated (red paths) based on user markings (yellow points); **c**: Inflated mesh

The strategy described above works well when the 3D model’s boundary entirely lies on the projection plane. However, this may not always be the case; for example, in Fig. 9, the broken part of the eggshell (highlighted) is not in the same plane as the rest of the boundary.

To handle these cases, we propose an approach that involves user input to identify such regions, and inflate them separately using the following steps:

- The user selects the start and endpoint of this boundary (say A and B), and any interior point C, to decide which path to take when going from A to B.
- The boundary to be inflated is then constructed to be the shortest path from A to B via C.
- This is a curve in R^2^, let γ ∈ [0, l] ➔ R^2^ represent the arc length parameterization, with parameter *s* and *l* represents the total length of the boundary to be elevated. Each point on γ is inflated by assigning it a height h: [0, l] ➔ R given by:
  2 $$ h(s)=k\ast \sqrt{{\left(\frac{l}{2}\right)}^2-{\left(s-\frac{l}{2}\right)}^2} $$

where *k* is a user-defined scaling variable, intuitively, this height field is semi-circular in shape, i.e., height is zero at A and B, and at a maximum when halfway between these points, it also ensures the continuous transitioning to the remaining boundary.

**Fig. 10:**
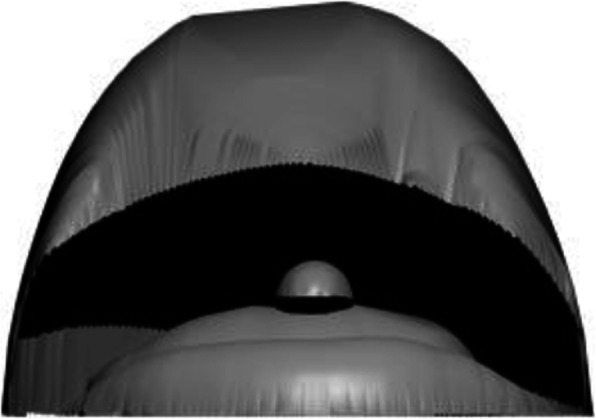
Top view of our boundary elevated model shown in Fig. 9c

**Fig. 11:**
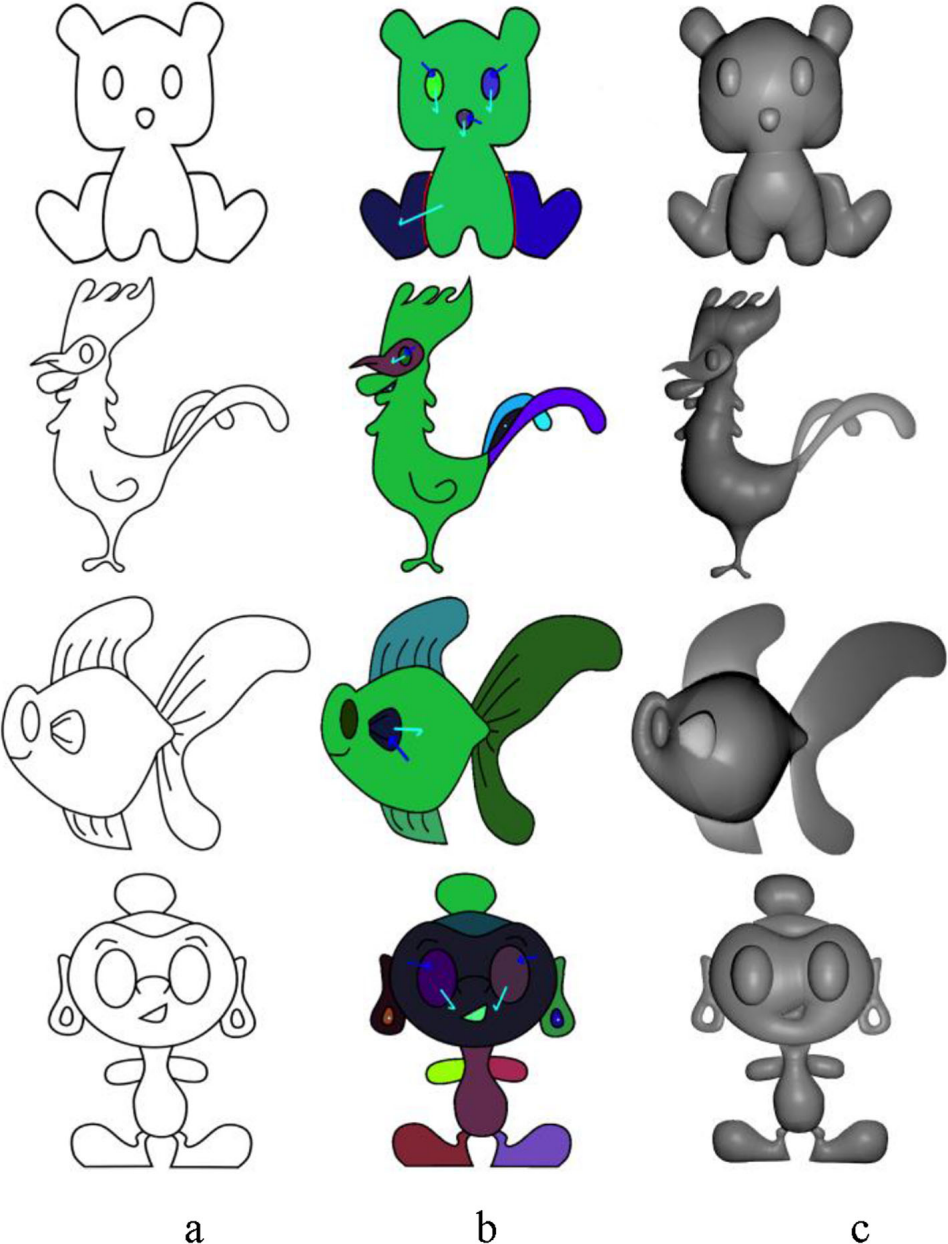
Results of our inflation procedure. **a**: Input sketch; **b**: Annotated regions; **c**: Result of our inflation procedure

Figure 10 shows the top view of the model in Fig. 9c, and it can be observed that the selected boundary is elevated. Some results generated using our inflation procedure are shown in Fig. 11. It should be noted that various features such as the object with holes (Buddha’s ear), sharp corners (points on the chicken’s comb), thin films (fin and tail of the gold fish), negatively elevated boundary (the back legs of the teddy bear) are generated using our simple approach.

The Delaunay-triangulation based inflation algorithm is easy to implement and conceptualize. Since we are directly manipulating the Delaunay-triangulation, hurdles such as computing Constrained Delaunay-triangulation without missing any important features in the input image, and pruning to find simplified symmetry axes, can be avoided. Also, since the system is intended for novice users, providing simple annotations (such as alignment and segmentation information) is better than complicated inputs such as bending strokes.

### Shading contour generation

Digitally shading a model is simplified because of the existence of various rendering algorithms. However, replicating the same procedure for shading in a physical medium is difficult due to less availability of the drawing medium shades and the complexity of the procedure involved. Further, many users are not experienced with the shading procedure. To tackle these problems, the objective in this phase is to create easily understandable guidelines that a novice user can follow. From kindergarten, we are familiar with filling a given boundary with a single color. Building on this familiarity, we observed that a sketch could be divided into a different set of boundaries such that each boundary can be filled with a single color. In this phase, the sketch is initially divided into such boundaries (iso-contours) based on the inflated 2.5D mesh, and then the color to be filled inside each boundary is computed.

### Iso-contour identification

**Fig. 12:**
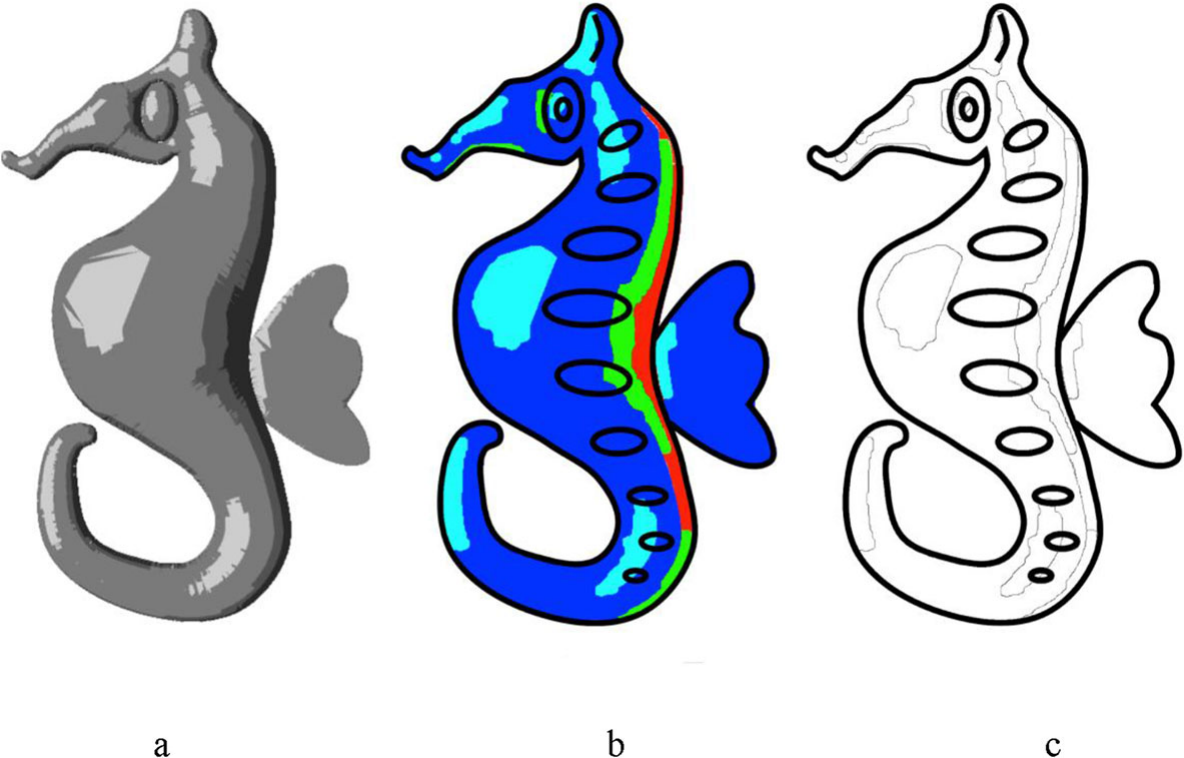
**a**: Toon shaded model; **b**: Identified regions; **c**: Generated iso-contours

**Fig. 13:**
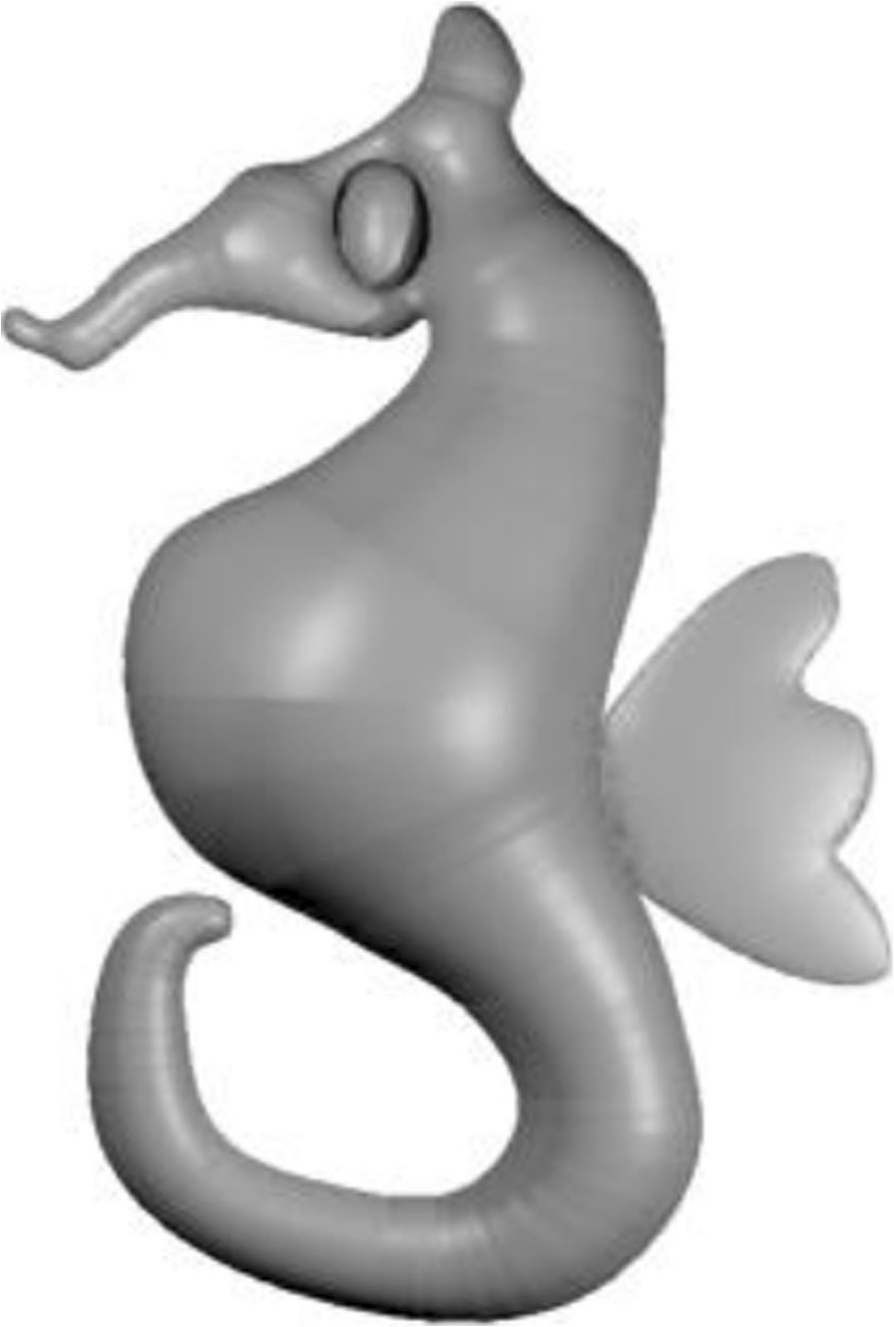
A sample inflated model

**Fig. 14:**
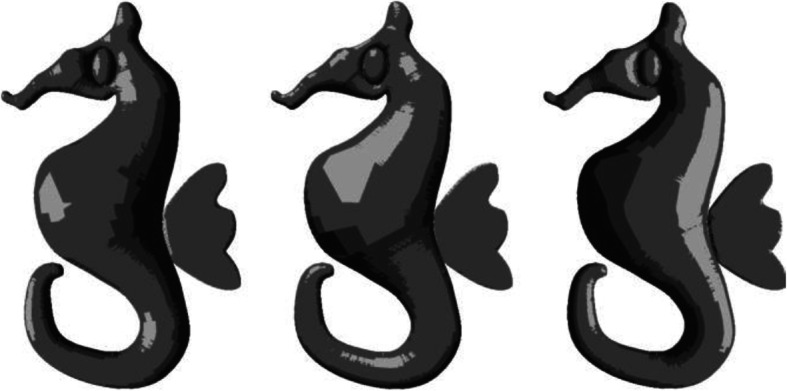
Toon shading with various light positions

**Fig. 15:**
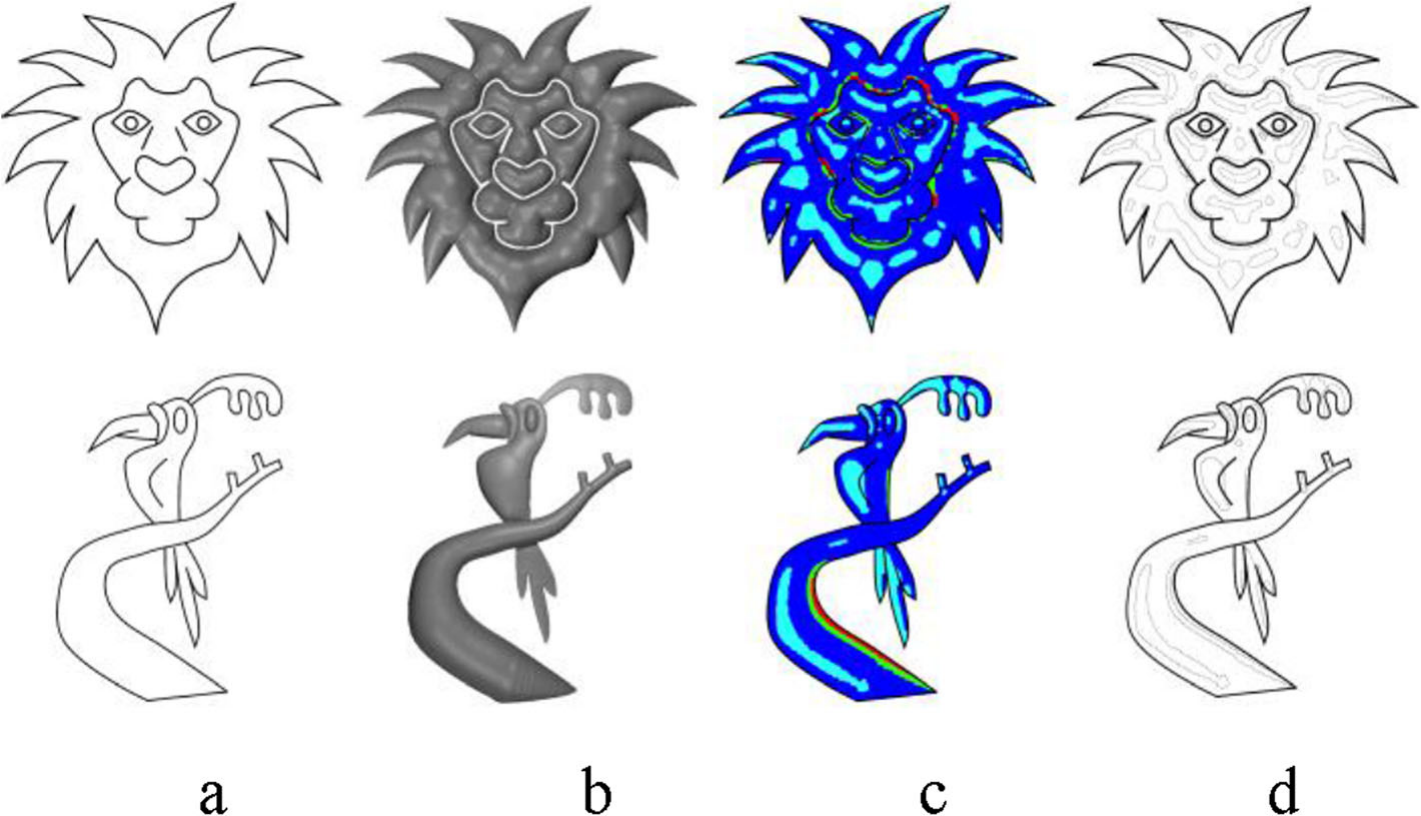
**a**: Input sketch; **b**: Inflated 2.5D model; **c**: Identified regions; **d**: Computed iso-contours

To divide the sketch into different boundaries, the pixels in the rendered 2.5D mesh must be classified based on the intensity. To achieve this, boundaries (iso-contours) are identified from the inflated model based on the toon shading of the 2.5D model. Iso-contours are computed by applying intensity-based thresholding on the toon-shaded model to divide the sketch into regions (for experimental purpose, we fixed the number of color levels of toon-shading at four). Opening followed by closing operations can be applied to individual regions, to remove the effect of small regions and to make shading easier. Figs. 12a-c show a sample toon shading (of a simple 2.5D mesh generated using our inflation algorithm as shown in Fig. 13), identified regions (cyan, blue, green and red show different regions with decreasing level of intensities) and computed iso-contours respectively. The user can also change the light direction and its intensity to create varieties of shading effects. Figure 14 shows the effect of toon shading for various light positions (For more information regarding the camera’s auto-exposure control, we refer the readers to refs. [28–31].). Some iso-contours generated using our algorithm, along with the corresponding sketch, inflated model, and identified regions are shown in Fig. 15.

### Color computation

Once the iso-contours have been computed, the colors to be filled in each boundary are identified using the following steps:

- The user digitally fills the sketch boundaries with some solid colors using a procedure similar to flood-fill.
- The solid color pixels are transformed into its dark and light shades according to the iso-contour it belongs to. A pixel is replaced by following the shades, meaning lighter, same, dark, and darkest shades if it lies in cyan, blue, green, and red areas (as shown in Fig. 12b), respectively.

Let the Red Green Blue colors of a pixel in a digitally colored sketch be (R_1_, G1, B1), the red component of the lighter shade is computed using the equation:

3 $$ {R}_l={\mathrm{R}}_1+\left(255-{\mathrm{R}}_1\right)\ast tf\kern0.50em $$

where *tf* is the tint factor. Using the same equation by replacing R_1_ by G_1_ and B_1_, the green and blue components of the pixel are computed respectively. As the value of *tf* increases, the tint becomes lighter and lighter and eventually becomes white when *tf* = 1. For experimental purposes, *tf* is set to 0.75. Similarly, the red component of the dark shade is computed using the equation:

4 $$ {R}_d={R}_1\ast \left(1- sf\right) $$

where *sf* is the shade factor. The R_1_ is replaced by G_1_ and B_1_ respectively to find the green and blue component of the pixel. As the value of *sf* increases, the shade becomes darker and eventually becomes black when *sf* = 1. For experimental purposes, *sf* is set to 0.4. The same equation is also used for the darkest shade computation by setting *sf* = 0.75.

**Fig. 16:**
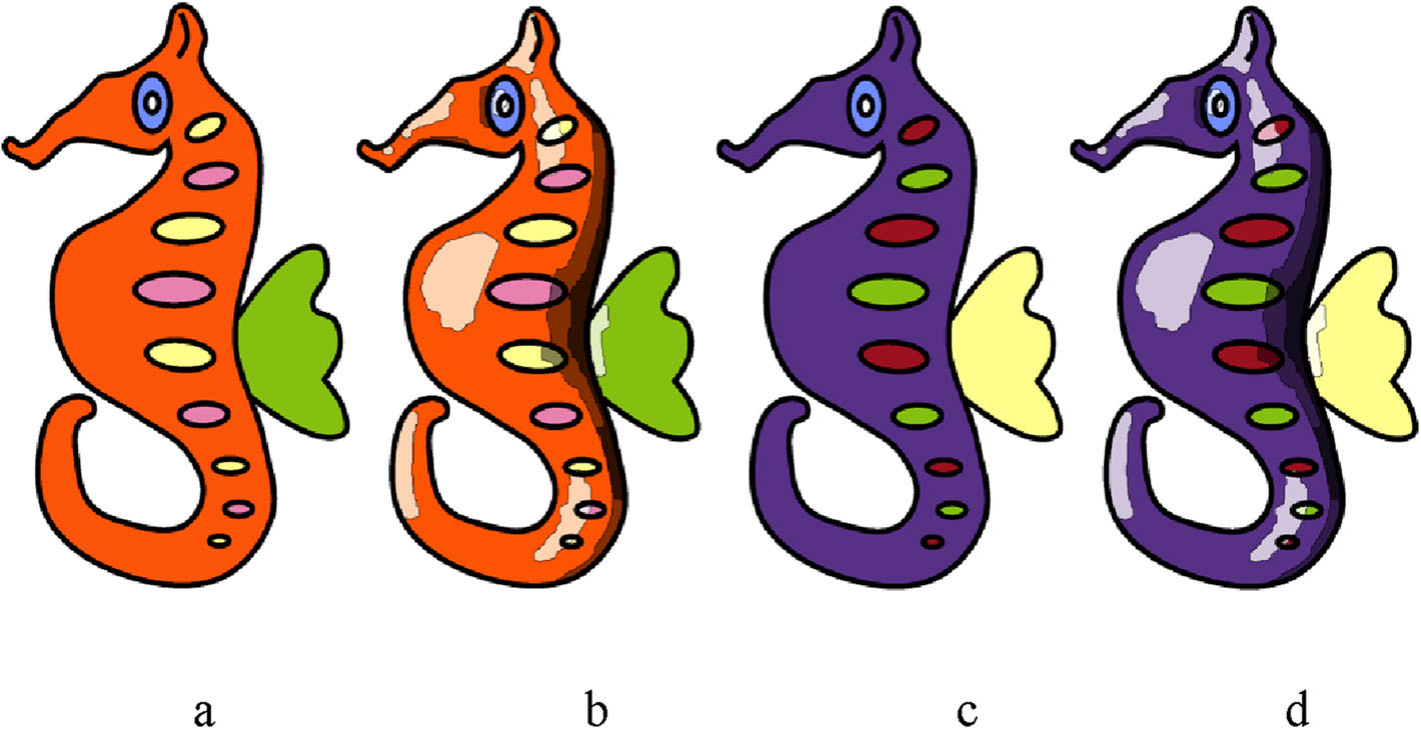
**a**, **c**: Sketches colored based on user annotations; **b**, **d**: Guideline images computed by our system

Figure 16 shows two sample colored sketches (based on user annotation) along with region-based shades computed by our system.

### Mixed reality interface

Once the iso-contours and shades are computed, this is delivered to the user. Since it is complicated for the user to translate the image and/or copy it directly to paper, we provide a mixed reality interface to simplify the task. Initially, the guidelines containing iso-contours and shades are copied to a guideline-image.

**Fig. 17:**
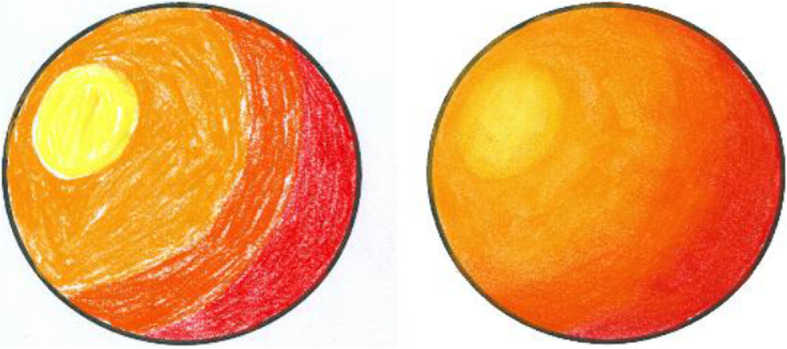
A sample shading before and after applying smudging

**Fig. 18:**
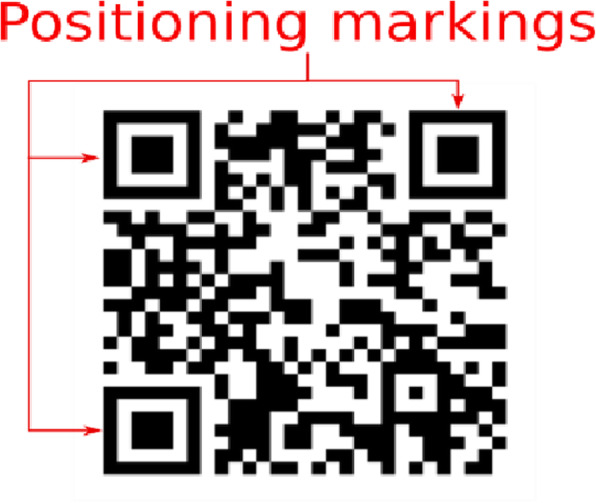
Positioning markings in a QR code

The guideline-image is then projected to the surface containing the sketch. This projection of iso-contours onto the sketch has various advantages, like the ability to quickly scale the iso-contours to fit sketches of various sizes, it is also cost-effective and easily available. Once the transparent iso-contours and respective colors are projected onto the screen, users are asked to fill each contour with the appropriate color. Once coloring is complete, according to their expertise level, they smudged boundaries to give a smooth transition of colors between contours. A sample shading before and after smudging the boundaries are shown in Fig. 17. To handle the mismatch between the size of the projected guideline-image and the original image (e.g., an image in a sketchbook), we provided a scaling tool in which the user can adjust the size of the guideline-image until it fits the sketch perfectly. Once the transparent guideline-image is projected onto the sketchbook, users are asked to fill each contour with the appropriate color. The setup works well if the position of the sketchbook is fixed. The coloring task becomes cumbersome if the sketchbook position is fixed. To overcome this uneasiness and to provide more flexibility, we must track the position of the sketch. To facilitate the tracking, we made use of the positioning markers of quick response (QR) codes (markers are shown in Fig. 18).

**Fig. 19:**
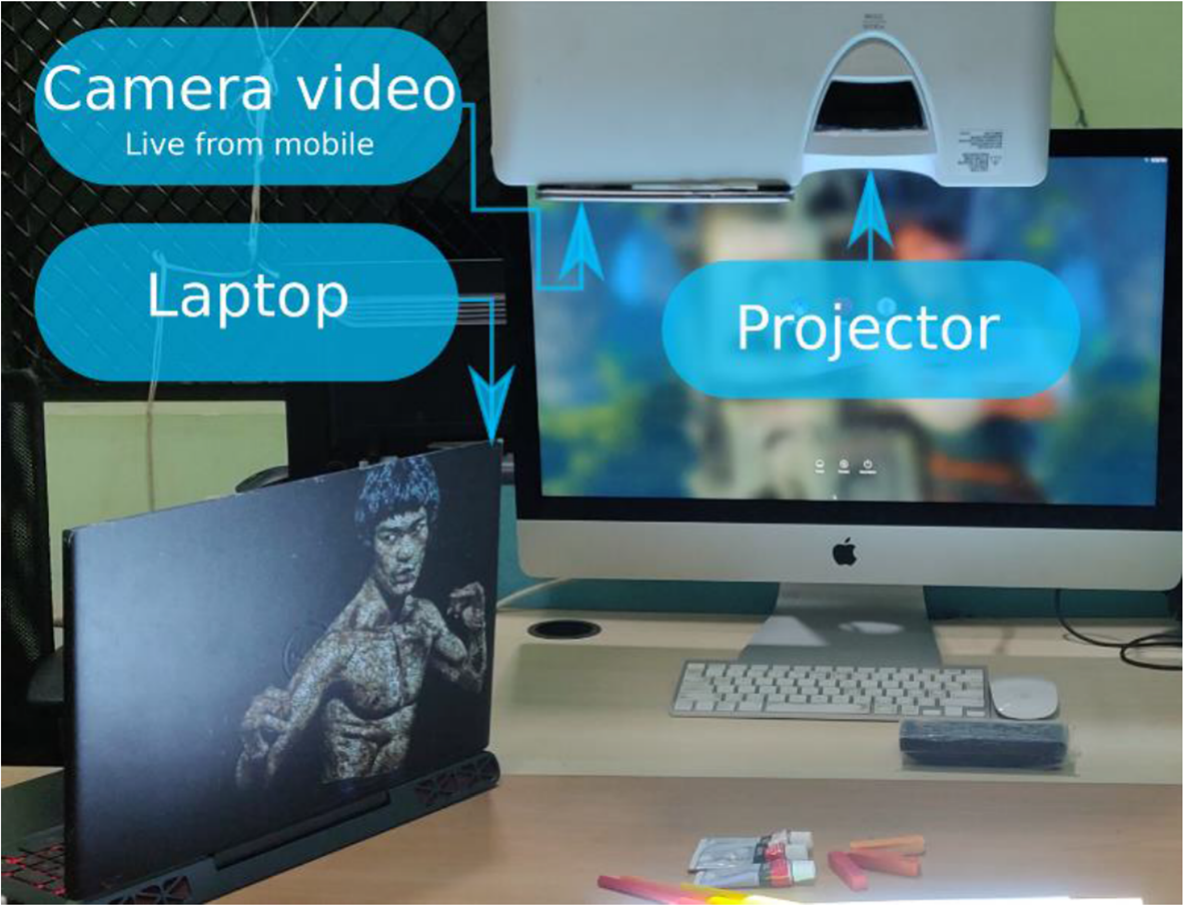
Overall hardware setup: The guideline-image is projected to the table with the help of a projector. Based on the position of the QR code in the captured video, appropriate transformations are applied on the guideline-image with the help of a computer


Our experimental setup consists of a low-cost projector, a mobile, and a laptop (Fig. 19). The need for a laptop can be avoided by creating a standalone mobile application. The projector is placed perpendicular to the drawing board with the mobile appropriately placed to capture the entire projection screen (both are kept in such a way that the QR codes are readable from the video feed). Once the hardware components are correctly placed, the guideline image is projected onto the shading surface. However, the user must keep the paper sketch stable to avoid the mismatch of alignment between the projected guideline image and the paper sketch. This is a major constraint that restricts user freedom. To overcome this issue, we proceeded with the following steps to facilitate real-time tracking of the paper sketch:

**Additional file 1: Video.**

**Fig. 20:**
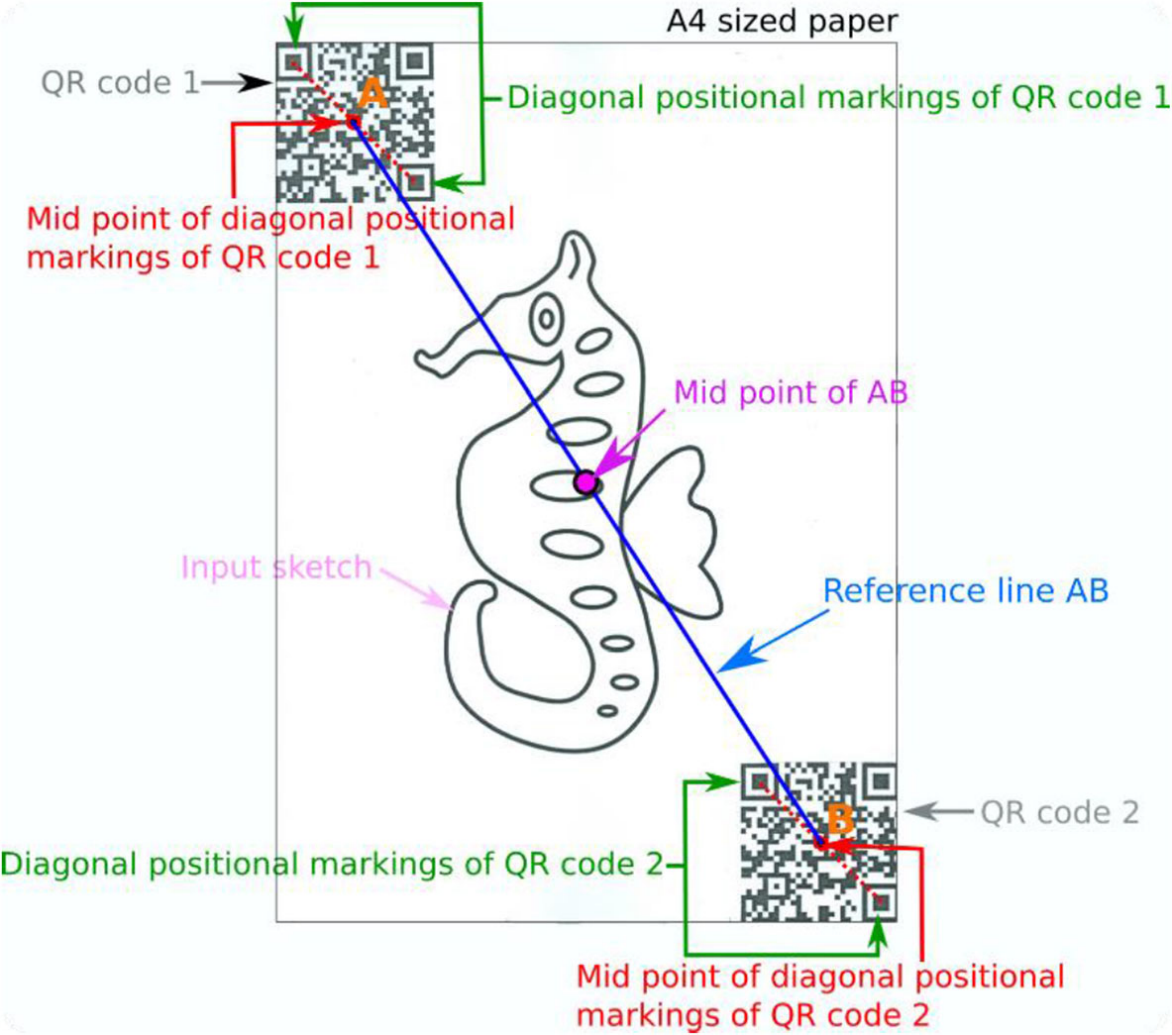
Information derived from a sample sketch in an A4 sheet along with QR codes fixed on its diagonal ends

- The guideline image is projected to the drawing surface.
- Since the resolution of the mobile and projector are different, the projection area alone is extracted from the video and resized to fit the projector resolution.
- The QR codes which are placed on the diagonal ends of the A4 sized sketch are located.
- With easy translate and scale functions, the user is asked to align the guideline-image to the paper sketch.
- The scaled and translated guideline image is resized based on the distance between QR codes.
- For each of the identified QR codes, the diagonal positional markings are identified by taking the two farthest markings.
- Once aligned, the reference line AB is constructed where A and B are the middle points of the identified diagonal markings of each QR code. Figure 20 shows a sample reference line on a sample sketch along with other related information.
- The position of A and B are monitored continuously until it deviates from the previous frame of video, and the guideline image is placed appropriately.

**Fig. 21:**
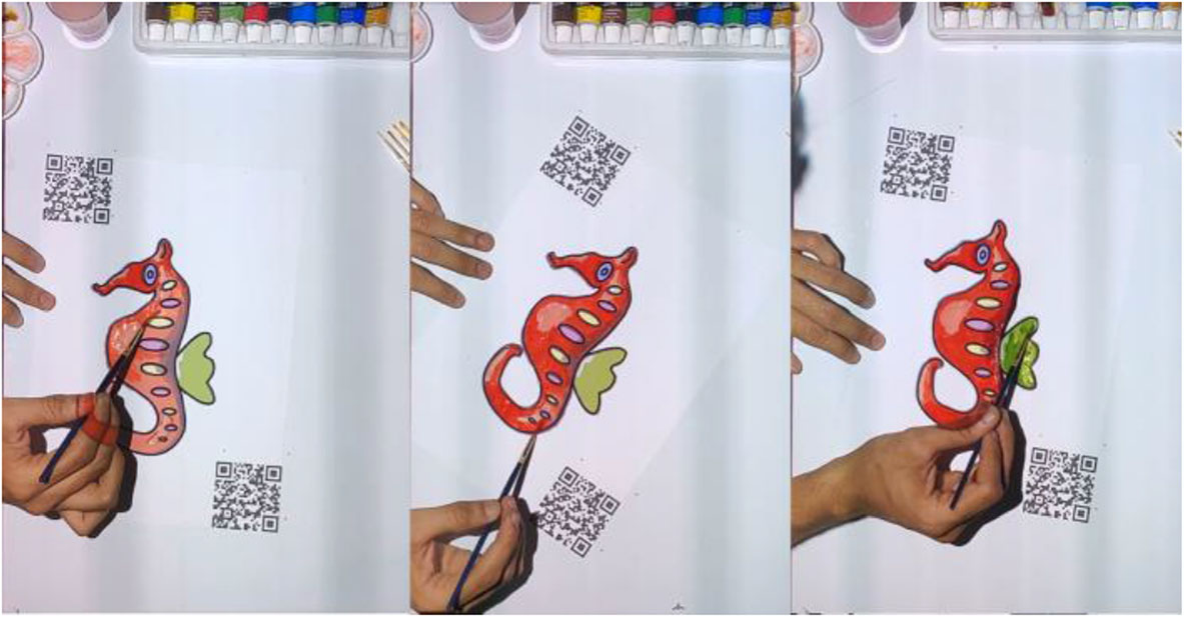
Some stills taken during the shading process of a paper sketch

**Fig. 22:**
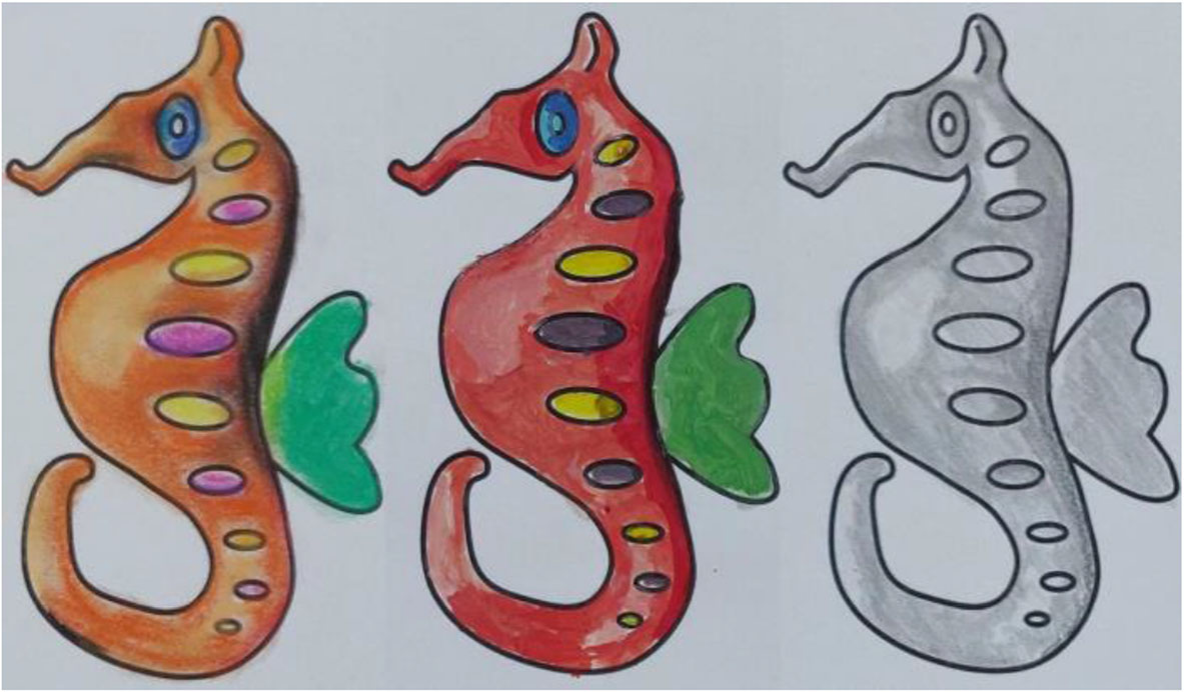
Shading given using different coloring mediums (pastel, water color and pencil respectively)

**Fig. 23:**
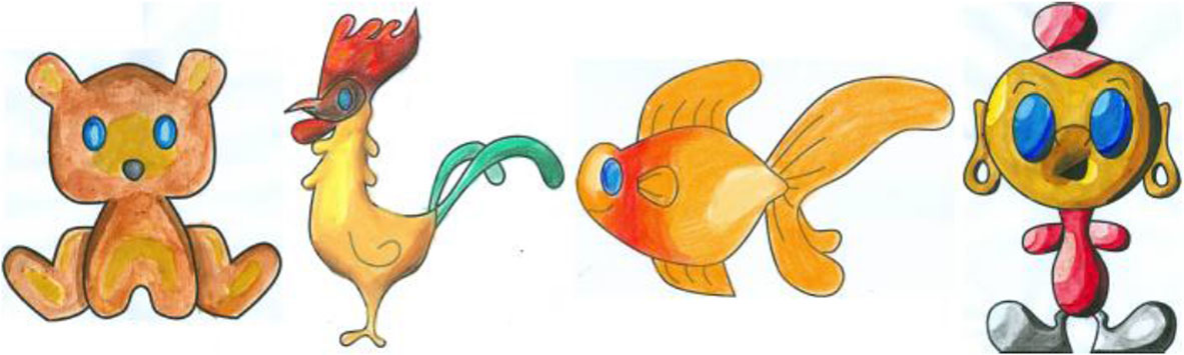
Some shaded paper sketches with the assistance of our system

**Fig. 24:**
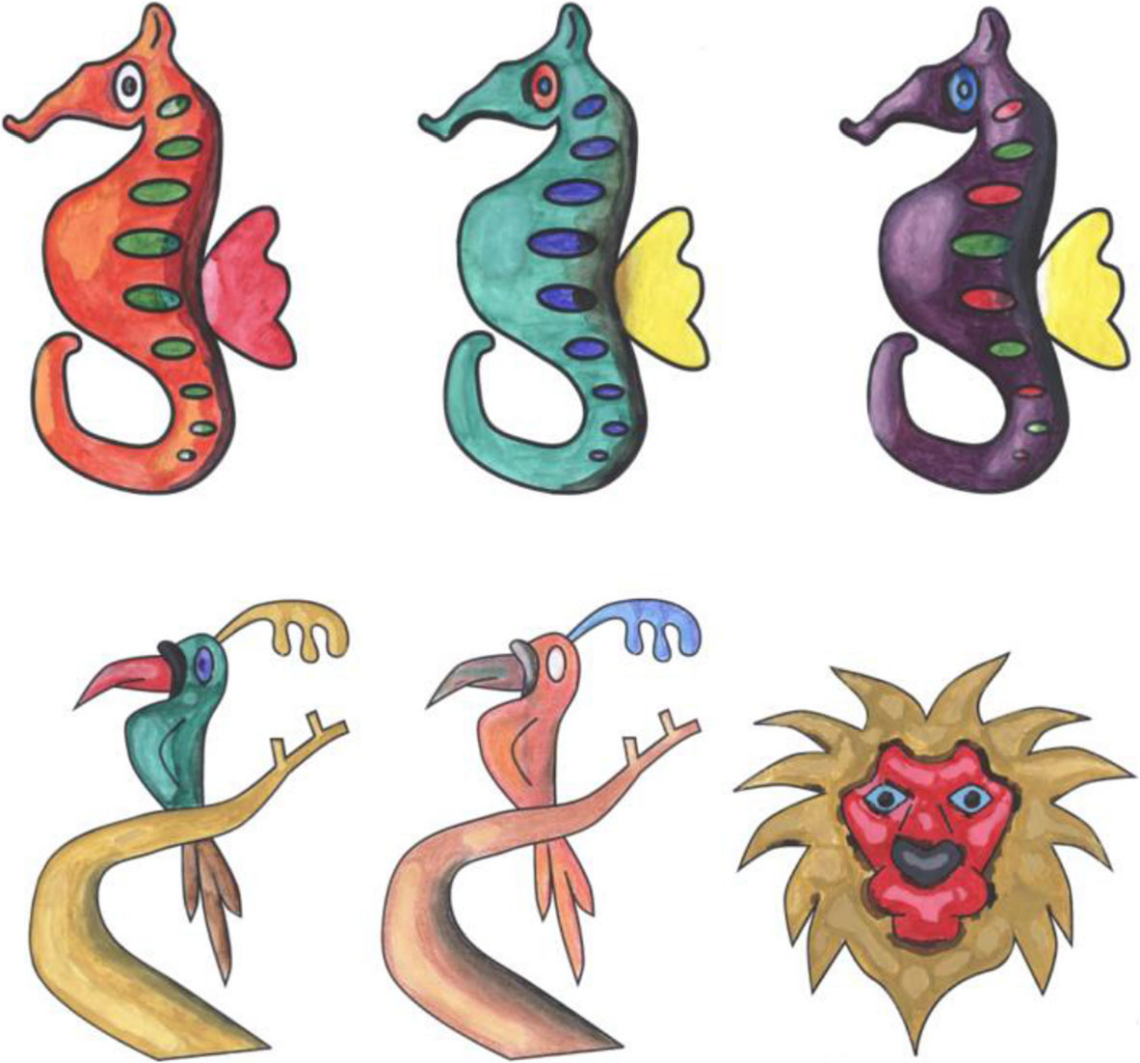
Shading done by various users with the help of our digital assistant

These steps make sure that the guideline image is always correctly aligned over the paper sketch, even if we move it. To avoid unnecessary transformations that might happen to the guideline image due to the missing QR code position (if either of the QR codes is blocked from the camera), transformations are applied only when both QR codes are visible in the video feed. During the user study, users were instructed to always keep the paper sketch inside the area lit by the projector and not to keep the hand/head over QR code for a long time. Since we are taking the video at a rate of 30 fps, we are not expecting the user to make a sudden 180 rotation to ensure our system works smoothly. Figure 21 shows a few instances from a user operating our system to create shades to a paper sketch. It can be observed that the guideline image is properly aligned over the paper sketch, even on different orientations. The system provides value irrespective of the shading medium used since the procedure is the same. Figure 22 shows a paper sketch colored with different mediums (pastel, water-color, pencil, respectively). Shading applied to the sketches shown in Fig. 11 using our system is illustrated in Fig. 23. The result of shading provided by various users with the help of our shading assistant is shown in Fig. 24.

**Fig. 25:**
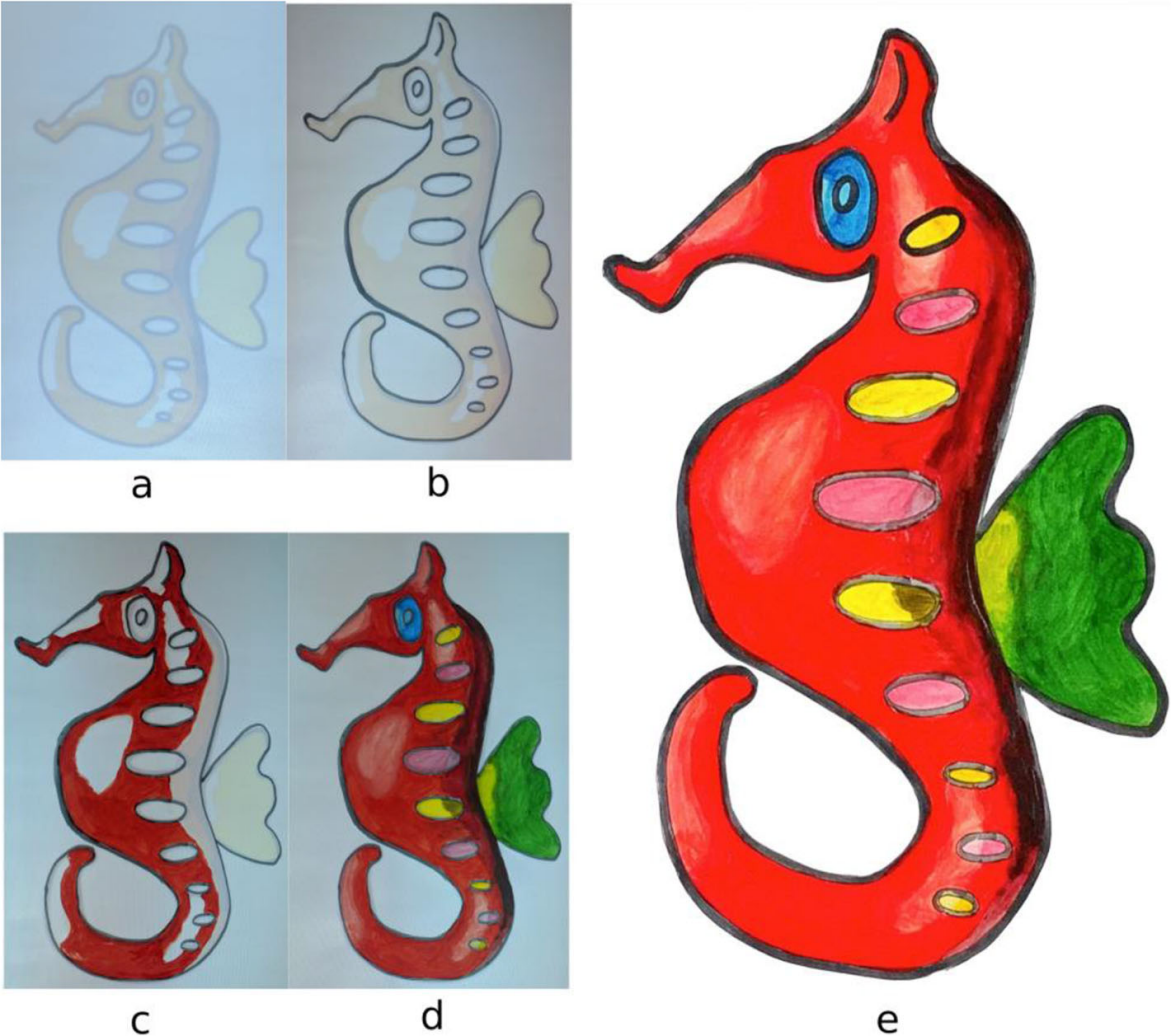
A large portrait created with the help of our system, a-e show various stages in the creation process and e shows the final result

Taking advantage of the scalability of the projector, by appropriately placing QR markers and the projector, we created a large portrait of size 565 mm × 720 mm with proper shading. This took approximately 2 h to complete. Figures 25a-d show various stages during the shading procedure and Fig. 25e shows the final shaded sketch.

## Conclusion, limitations and future works

We have demonstrated a new approach to assist users in shading a paper sketch (without the availability of shading information). The proposed system is targeted towards unskilled users to provide an artistic feel to the sketch. Our system initially creates a 2.5D representation from the sketch with the help of a few user-generated strokes and is further used for computing iso-contours and appropriate colors. We found that the proposed system increased the ability of users to create artistic shading with 3D look-alike cues. Our system can also be used for creating large portraits (Fig. 25).

Even though our system is easy to use, it has a few limitations. For example, we require some user interaction to create a 2.5D representation. Further, we assume the shapes have elliptical cross-sections (which is a limited subset). One of the interesting future directions could be to generalize 2.5D modeling by including varying cross-sectional objects. Though our system provides the color to be filled in each iso-contour, it does not provide information about the color combinations to be used to create the shade, which is sometimes a difficult task for first time users. Thus, the system could be improved by implementing this detail.

## Data Availability

Please contact the corresponding author for data requests.

## Abbreviations

2D: 2-Dimensional
3D: 3-Dimensional
2.5D: 2.5-Dimensional
DT: Delaunay triangulation
QR code: Quick response code

## References

[1] Laviole J, Hachet M (2012) PapARt: interactive 3D graphics and multi-touch augmented paper for artistic creation. Paper presented at the 2012 IEEE symposium on 3D user interfaces, IEEE, Costa Mesa, 4-5 march 2012. 10.1109/3DUI.2012.6184167

[2] Shilkrot R, Maes P, Paradiso JA, Zoran A (2015). Augmented airbrush for computer aided painting (CAP). ACM Trans Graph.

[3] Rivers A, Adams A, Durand F (2012). Sculpting by numbers. ACM Trans Graph.

[4] Liu LJ, Ceylan D, Lin C, Wang WP, Mitra NJ (2017). Image-based reconstruction of wire art. ACM Trans Graph.

[5] Song P, Wang XF, Tang X, Fu CW, Xu HF, Liu LG (2017). Computational design of wind-up toys. ACM Trans Graph.

[6] Li XY, Shen CH, Huang SS, Ju T, Hu SM (2010). Popup: automatic paper architectures from 3D models. ACM Trans Graph.

[7] McCrae J, Umetani N, Singh K (2014). FlatFitFab: interactive modeling with planar sections. Paper presented at the 27th annual ACM symposium on user interface software and technology.

[8] Agrawal H, Umapathi U, Kovacs R, Frohnhofen J, Chen HT, Mueller S, et al (2015) Protopiper: physically sketching room-sized objects at actual scale. Paper presented at the 28th annual ACM symposium on user interface software & technology, ACM, Charlotte, 11-15 November 2015. 10.1145/2807442.2807505

[9] Igarashi Y (2011). Deco: a design editor for rhinestone decorations. IEEE Comput Graph Appl.

[10] Igarashi Y, Mitani J (2014). Weavy: interactive card-weaving design and construction. IEEE Comput Graph Appl.

[11] Igarashi Y, Igarashi T, Mitani J (2016). Computational design of iris folding patterns. Computat Vis Media.

[12] Prévost R, Jacobson A, Jarosz W, Sorkine-Hornung O (2016). Large-scale painting of photographs by interactive optimization. Comput Graphics.

[13] Clark A, Dünser A, Grasset R (2011) An interactive augmented reality coloring book. Paper presented at the SIGGRAPH Asia 2011 emerging technologies, ACM, Hong Kong, 12-15 December 2011. 10.1145/2073370.2073394

[14] Magnenat S, Ngo DT, Zünd F, Ryffel M, Noris G, Rothlin G (2015). Live texturing of augmented reality characters from colored drawings. IEEE Trans Vis Comput Graphics.

[15] Parakkat AD, Joshi SA, Pundarikaksha UB, Muthuganapathy R (2017) Sketch and shade: an interactive assistant for sketching and shading. In: proceedings of the symposium on sketch-based interfaces and modeling, ACM, California, Los Angeles, 29-30 July 2017. 10.1145/3092907.3122799

[16] Crayola Color Alive (2019). http://www.crayola.com/. Accessed 12 Nov 2019

[17] Chromville (2019). https://chromville.com/. Accessed 12 Nov 2019

[18] Color and play (2019). http://www.onlycoloringpages.com/. Accessed 12 Nov 2019

[19] Feng LL, Yang XB, Xiao SJ (2017) MagicToon: a 2D-to-3D creative cartoon modeling system with mobile AR. Paper presented at 2017 IEEE virtual reality, IEEE, Los Angeles, 18-22 march 2017. 10.1109/VR.2017.7892247

[20] Flagg M, Rehg JM (2006) Projector-guided painting. Paper presented at the 19th annual ACM symposium on user interface software and technology, ACM, Montreux, 15-18 October 2006. 10.1145/1166253.1166290

[21] Anjyo KI, Wemler S, Baxter W (2006) Tweakable light and shade for cartoon animation. Paper presented at the 4th international symposium on non-photorealistic animation and rendering, ACM, Annecy, 5-7 June 2006. 10.1145/1124728.1124750

[22] Todo H, Anjyo KI, Baxter W, Igarashi T (2007). Locally controllable stylized shading. ACM Trans Graph.

[23] Hudon M, Pagés R, Grogan M, Ondřej J, Smolić A (2018) 2D shading for cel animation. Paper presented at the joint symposium on computational aesthetics and sketch-based interfaces and modeling and non-photorealistic animation and rendering, ACM, British Columbia, 17-19 august 2018. 10.1145/3229147.3229148

[24] Panotopoulou A, Paris S, Whiting E (2018). Watercolor woodblock printing with image analysis. Comput Graphics Forum.

[25] Igarashi T, Matsuoka S, Tanaka H (1999) Teddy: a sketching interface for 3D freeform design. Paper presented at the 26th annual conference on computer graphics and interactive techniques, ACM, Los Angeles, July 1999. 10.1145/311535.311602

[26] Borosán P, Jin M, DeCarlo D, Gingold Y, Nealen A (2012). RigMesh: automatic rigging for part-based shape modeling and deformation. ACM Trans Graph.

[27] Methirumangalath S, Parakkat AD, Muthuganapathy R (2015). A unified approach towards reconstruction of a planar point set. Comput Graphics.

[28] Liang JY, Qin YJ, Hong ZL (2007) An auto-exposure algorithm for detecting high contrast lighting conditions. Paper presented at 2007 7th international conference on ASIC, IEEE, Guilin, 22-25 October 2007. 10.1109/ICASIC.2007.4415733

[29] Su YH, Lin JY, Kuo CCJ (2016). A model-based approach to camera's auto exposure control. J Vis Commun Image Represent.

[30] Su YH, Kuo CCJ (2015) Fast and robust camera’s auto exposure control using convex or concave model. Paper presented at 2015 IEEE international conference on consumer electronics, IEEE, Las Vegas, 9-12 January 2015. 10.1109/ICCE.2015.7066300

[31] Cho M, Lee S, Nam BD (1999) Fast auto-exposure algorithm based on numerical analysis. Paper presented at SPIE 3650, sensors, cameras, and applications for digital photography, SPIE, San Jose, 22 march 1999. 10.1117/12.342853

